# Do Tournament Incentives Matter for CEOs to Be Environmentally Responsible? Evidence from Chinese Listed Companies

**DOI:** 10.3390/ijerph19010470

**Published:** 2022-01-01

**Authors:** Sajid Ullah, Farman Ullah Khan, Laura-Mariana Cismaș, Muhammad Usman, Andra Miculescu

**Affiliations:** 1 School of Economics and Management, Xi’an University of Technology, Xi’an 710048, China; sajidkhan6695@stu.xaut.edu.cn; 2 School of Management, Xi’an Jiaotong University, Xi’an 710049, China; 3 Faculty of Economics and Business Administration, West University of Timisoara, 300006 Timisoara, Romania; andra.miculescu@e-uvt.ro; 4 School of Accounting, Nanjing Audit University, Nanjing 210017, China

**Keywords:** green innovation, CEO tournament incentive, tournament theory, state-owned enterprises, China

## Abstract

Relying on tournament theory and environmental management research, we examine how CEO tournament incentives induce top executives to invest more in green innovation. Using a sample of Chinese listed companies from 2010 to 2016, we find evidence that CEO tournament incentives are positively associated with green innovation. In addition, we find that a positive relationship between CEO tournament incentives and green innovation is stronger in state-owned enterprises than in non-state-owned enterprises. These results support tournament theory, which proposes that better incentives induce top executives’ efforts to win the tournament incentives, and such efforts are subject to fiercer competition among employees, which improves firms’ social and financial performance. Moreover, our findings have implications for policy makers and regulators who wish to enhance environmental legitimacy by providing tournament incentives to top executives.

## 1. Introduction

The recent stratospheric emphasis on incentivizing executives for achieving social goals has caught scholars’ attention. Companies concerned about environmental degradation are more likely to correlate executive compensation to environmental performance, recognizing that management should be rewarded for the higher risk associated with long-term goals (i.e., environmental performance) [1]. Indeed, some firms connect CEO compensation to environmental performance, recognizing that if they connect CEO compensation and environmental performance, they can effectively reduce environmental degradation in polluting industries [1]. As developing countries’ environmental quality are extremely low, moreover, these countries are likely to face green innovation adoption challenges [2]; therefore, researchers are looking for drivers to promote green innovation in underdeveloped countries [3]. Because investment in green innovation is highly risky, and the payback period is long [4,5], the agency costs attached to such innovation are likely to be high, and designing an incentive package to induce innovation is specifically demanding [6].

Prior researchers in the field of environmental management sought to determine the factors that drive green innovation. In doing so, it was established that internal drivers are the most fascinating factors affecting sustainability performance [7,8,9]. The top management is a crucial internal factor for adopting green initiatives to improve social and environmental performance [10,11,12,13] because the top managers are accountable for firms’ ethical actions, such as ensuring the implementation of sustainability initiatives [14,15,16]. Being a powerful member of a top management team, a chief executive officer (CEO) pledges with his/her strong will to focus on all stakeholders, including the customers, shareholders, society, and environment at large [17,18]. Some studies asserted that green practices in firms cannot be entrenched without the top management’s (CEOs) support [19,20].

Therefore, research on top management has bolstered an effort to disclose what determines and motivates executives to be environmentally responsible. Because of their individual qualities, which provide them with a high level of satisfaction in such endeavors, top executives engage in environmentally friendly activities [21,22]. The top executives tend to deploy firm resources for the enhancement of their own narrow financial benefits at the cost of the company’s long-term objectives, and, hence, are hesitant to invest in environmental projects that help organizations in the long run [1,23]. In this vein, scholars have largely focused on executives’ traits and their effect on green performance, such as a CEO’s power, [24], political affiliation [25], duality [26], gender [27], hubris [18], education and tenure [28] as well as compensation [1]. Based on upper echelons theory, research has found a link between CEO gender and corporate social performance. Many academics contend that female CEOs are more concerned with CSR-related topics than male CEOs [29,30]. According to the authors, women are more society- and environment-oriented, whereas male managers are more self-oriented, based on the concept of social role theory. The differences in the CEOs’ careers have varied social outcomes [31]. Because young managers must deliver good observable results to the market, the authors suggest, they are more inclined to adopt measures that focus on short-term observable outcomes and less likely to increase social- or environmental-related field operations. Unlike younger executives, elderly CEOs are not under as much market pressure as their younger counterparts; therefore, they are more motivated to care about corporate social performance. Several studies using upper echelons theory have found a correlation between CEO education and corporate social performance. Huang [32] conducted research in which the educational specialization of CEOs was shown to be related to the CSR performance of companies. Similarly, Lewis, Walls, and Dowell [28] discovered that managers with MBA degrees are more likely than other enterprises to disclose environmental information, but peers with law degrees are less likely. However, our research goes beyond the existing literature on the demographics of CEOs by exploring whether tournament incentives motivate chief executives to spend more on environmentally friendly innovations.

The connection of CEO remuneration to social and environmental performance (e.g., greenhouse gas reduction targets, staff training in green practices, and adherence to moral principles in developing countries)—is a modern phenomenon in corporate governance. Many companies have bound compensation structure to sustainability performance; for instance, Xcel Energy implies that 75 percent of its incentives are still based on profits per share growth, but the other 25% are based on ecological footprint and greenhouse gas emissions reductions. Likewise, Intel connects CEO pay to corporate environmental targets, such as product energy efficiency, carbon emissions and power consumption savings, as well as improved environmental leadership reputation [33]. Superior social and environmental performance is often the result of long-term efforts that demand a long-term approach [34,35]. Firms may gain trust and acquire the social license to operate by actively interacting with local communities and contributing to their well-being over time. Similarly, businesses may reduce their environmental impacts by investing in renewable technology, sustainable energy, and other green initiatives that will take time to yield tangible results. We anticipate that incentivizing managers based on social and environmental performance goals will attract them to focus on longer-term initiatives, leading them to embrace a longer time horizon. As a result, they are less likely to ignore valuable long-term stakeholder initiatives, improving the firm’s worth. Key stakeholders’ pressure might encourage CEOs to practice quality management in order to maintain their market reputation. As a result, the CEO’s quality management efforts are strengthened by fulfilling the needs of the major stakeholders. Moreover, winning an industry award improves a CEO’s reputation while also increasing a firm’s credibility in the eyes of its key stakeholders [36,37]. Higher levels of reputation, as well as primary and secondary stakeholder pressures, are linked to higher levels of quality management, which leads to environmental innovation [38]. As a result, when managers are properly rewarded, they are more likely to take steps to reduce their environmental impact. They could, for example, reduce pesticide use, minimize energy, implement a recycling system, involve their employees in community clean-ups and environmental protection efforts, upgrade their facilities to prevent oil spills and other environmental disasters, construct green buildings, or switch to renewable energy and clean fuels. We expect firms’ emissions to diminish as a result of such measures [33].

The recent trend in CEO tournament incentives suggests that tournament incentives are the pay gap between CEO and other executive pay and likely a powerful steward that may prompt executives’ performance [39,40,41,42,43]. It is obvious that participants in the competition deploy their full potential if pay is based on a tournament scheme [44]. Specifically, the tournament theory posits that the variance in pay between CEOs and other executives leads to a fiercer competition that exerts more efforts to improve firms’ performances for winning the contest [45,46]. Thus, the motivation of this study takes its philosophical roots from tournament theory, which seeks to understand the mechanism by which incentive packages induce green performance. As top executives are assessed on the basis of their relative performance, rather than absolute performance, our study predicts that tournament incentives will motivate CEOs to be more environmentally responsible in response to huge incentives gaps.

Our study contributes in two significant aspects. First, this study extends from prior research on the internal determinants of green performance [8,9,12,13,47] by exploring chief executive tournament incentives as an important determinant of green innovation performance. We submit that CEO tournament incentives positively affect firms’ green innovation, suggesting that a compensation gap drives CEOs to be green by investing more in green innovation. Second, the situation becomes particularly vital when we demonstrate this within the Chinese context. China is a transition economy designed to pool resources of land, capital, and human resource in emerging markets. Simultaneously, the state plays a monopolistic role, in terms of asset, policy, and law execution [48], and runs SOEs to exercise financial and social activities [49]. As the institutional environment of China is distinct in nature [50,51], former researchers recommended that effective corporate governance must acknowledge the institutional environment wherein corporate entities maneuver [52,53]. However, in the case of China, most of the listed firms are state-owned enterprises, and the state as a major shareholder gives more priority to social, environmental, and political goals [54,55]. Thus, we contribute by exploring whether the impact of chief executive tournament incentives on green innovation differs among state firms and non-state firms. Our study also finds that the positive impact of tournament incentive is more noticeable in state enterprises than it is in non-state enterprises.

## 2. Literature Review

### 2.1. CEO Tournament Incentives and Green Innovation

A firm’s participation in social activities is influenced by a variety of factors at firm level, and, among them, the top executives’ incentives play a central role in motivating management efforts. The impact of executive pay disparity on a company’s social and financial consequences has sparked open discussion [56,57]. According to certain academics, CEO compensation is positively related to environmental performance [1,58]. A stream of studies suggests that pay gap can be an effective motivator and stimulate pay rivalry, which motivates executives to exert effort for involvement in long term projects—such as R&D projects [59,60,61,62]. More related to social and environmental performance, previous research highlights that executives are awarded higher incentives for social and environmental endeavors that have many significant implications including personal reputation, career enhancement, stakeholder satisfaction, and organizational legitimacy [63,64,65,66]. The previous studies suggested that environmental process innovation, business analytics, and environmental product innovation have a significant impact on the green competitive advantage of the companies [67,68]. Corporate social responsibility is exemplified by environmental innovation. Significant eco-innovation may have a favorable influence on a firm’s reputation and political links, resulting in intangible assets and the ability to raise capital. As a result, substantive eco-innovation is linked to the long-term success of businesses, and businesses should invest in substantive eco-innovation in order to obtain resources [69].

Conversely, other authors documented a negative and weak relationship between executive compensation and environmental and social performance [70,71,72,73], as investment in social objectives could be a financial burden on a firm’s financial performance. Furthermore, stakeholder mismatching happens when stakeholders who receive an advantage from environmental reputation (e.g., the general community) are not the same individuals who assess a firm’s performance (e.g., stockholders) [74]. In the executive pay structure, the same paradox exists. The large pay gaps between executives and other employees leads to a negative impression that discourages the collective efforts of employees, which is detrimental to innovation [75,76].

However, in this research, we look at how CEO tournament incentives influence green innovation in China, where the regulatory structure embodies the intriguing features of an emerging and transition economy [77,78], and where the general public is becoming completely conscious of the worsening air quality and the dangers posed by industrial pollution [79,80]. As the environmental standards are lower in China compared to developed countries, and environmental rules and their enforcement are notoriously weak [81,82], we posit that such tournament incentives can encourage top managers to harvest green practices.

The interesting logic behind tournament incentives is that a CEO is sitting on the top ladder rung of the firm, where there are no chances of further promotion; thus tournament incentives can be considered as a catalyst to trigger a CEO’s effort in order to invest in social projects [55], while other senior executives exert effort for promotion-based and performance-based incentives [83,84]. If senior executives receive a promotion to the CEO position, they receive modest pay and incentives, and such compensation schemes enhance their motivation level for winning prizes and corporate performance [45,85]. Firms that believe that an organization’s competitive environment encourages talent will prefer high compensation, as assumed by tournament theory [86,87]. According to this theory, compensation based on position is an effective reward method for motivating employees [45]. Previous researchers demonstrate that higher tournament incentives tend to increase wide-spread firm activities, including research and development that translates into firm performance [88,89]. The perceived value of tournament incentives is that they motivate managers to put in more effort, which leads to more innovative performance. Consistent with this conjecture, Shen and Zhang [90] find evidence in U.S. public firms that tournament incentives strengthen the efficiency of firms’ innovations. This implies that managers usually respond actively to tournament incentives to achieve better results.

A group of studies has shown that one manifestation of the greater effort made by competing against other executives is risk-taking [91,92,93]. For instance, in professional sports competitions, there is only one winner who receives a high prize; nonetheless, all other players adopt an aggressive risk-taking approach since they have nothing to lose and are hoping to gain great results by taking risks (Grund et al. 2013). Similarly, a higher tournament incentive brings over investment in risky innovative projects without calculating the potential benefits that might have a drastic effect on a firm’s innovative capability [94]. Goel and Thakor [95] also find that executives can increase the probability of winning a tournament by plunging into excessive risky projects. The evidence on how tournament incentives affect executives’ risk-taking behaviors and innovations, however, remains relatively scant in organizational governance literature [90].

Drawing on tournament theory, we hypothesize that CEO tournament incentives are a driving force that triggers green innovation because investment in green projects relies on the CEOs’ decisions, and these incentives will be a stepping stone towards the long-term stability of an organization by achieving higher environmental performance. Overall, this discussion leads us to the following hypothesis:

**Hypothesis** **1** **(H1).** *Tournament incentive is positively related to green innovation*.

### 2.2. The Moderating Role of Ownership Structure

Next, we predict a higher correlation between a CEO tournament incentive and green innovation in state-owned businesses than in non-state-owned companies. Ownership type is an environmental catalyst for green innovation that influences the tendency towards going green. The government, as a major shareholder, tightly controls the financial and non-financial resources for innovation [96].

In a developing market (such as China), the government seems to be the most important institution, wielding enormous influence over finite resources and enacting policies to determine enterprises’ goals in a competitive market [97]. The government is considered a significant stakeholder to secure the uncertain risk involved in green innovation because, if the state does not provide enough support in policies, it is tough for businesses to generate long-term growth [98]. China is a transition economy characterized by public and private ownership, where the regulatory framework illustrates the features of an emerging market [77]. In particular, the Chinese government motivate firms towards environmental legitimacy [99], and the government acts as a stewards of SOEs to implement its social and economic agenda [49]. SOEs are often large, whereas private businesses are typically small. As a result, disparities in CSR that appear to be caused by ownership may instead be due, instead, to the size of the company. Furthermore, CSR assessments differ from one another since various items are used in different research. Faced with market transition, increased competition, and crucial trade frictions, Chinese private firms failed to compete with SOEs, foreign-owned enterprises, and multinational enterprises, especially due to their tiny size, inexperienced management, and poor technology [100].

According to institutional theory, companies also improve their environmental legitimacy because of external stakeholder pressure. In such a scenario, a firm’s establishment of a connection with the government is one significant approach to create legitimacy [101]. Major Chinese state-owned firms solely rely on the government for receiving opportunities involved in new product development; thus, such enterprises avail themselves the benefits of approving patents and the financial and other resources required for innovation. As managers of state-owned enterprises have easy access to government support, policy information and other guidelines, this may also affect the firms’ likelihoods towards investment in environmental innovation, which further provides a signal of organizational legitimacy [102,103]. Because CEOs build social ties with the government in a way that strengthens recognition as a government representative, they obtain more government support and resources for combating environmental risk. Farag et al. [104] found that SOEs are more motivated to invest in socially responsible activities than NSOEs and are more conscious about environmental protection. Such evidence echoes that of Usman et al. [105] and Huang et al. [106], who identified Chinese listed firms that politically led CEOs to promote more green innovation in SOEs than NSOE. These CEOs established diplomatic relations with the government, which provides an edge to access information, environmental policies, and state support. In response, firms may face more pressure for environmental legitimacy.

Taken together, top executives in state enterprises are more motivated to make decisions in favor of the state, which places them in a better position to win tournaments or promotion rewards [107]. Ethical leadership of state enterprises shows a commitment to enhance the reputation of a firm using the investment in CSR because of state regulatory due diligence [108]. The executives in SOEs are assessed on the basis of social and financial performance; thus, social activist CEOs strive to engage more in green innovation for image-building in order to obtain government privileges and win incentives, as CEOs of state-owned firms do not face these financial barriers. Furthermore, CEOs of Chinese state-run firms are usually supervised, hired, and promoted through political interference, and their pay is compressed in alignment with state interests. As a result, SOE board members and management are highly incentivized to make decisions that benefit the state by putting social goals ahead of economic objectives [61,109]. Moreover, the Chinese government offers grants to state firm top executives [110] in return for legitimate business actions [111]. Consequently, sound tournament incentives will induce the state-owned enterprise CEOs to invest more in green innovation. We draw the hypothesis that the influence of CEO pay incentives on green innovation would be more evident in state enterprises than in non-state enterprises. Thus, we formulate the following hypothesis:

**Hypothesis** **2** **(H2).** *The positive impact of CEO tournament incentives on green innovation is more pronounced in SOEs than in non-SOEs*.

## 3. Methodology

### 3.1. Data Sources and Sample

This study used a panel dataset of Chinese A-share companies listed on the Shanghai and Shenzhen stock exchanges for the period ranging from 2010 to 2016. The data relating to green innovation is obtained from the National Intellectual Property Administration (NIPA) database. The NIPA database provides summary details for all information about innovation type, title, application date, applying firm or person, and a short summary of the patent. We also used the enterprises’ annual reports and China Stock Market and Accounting Research (CSMAR) to collect data on the independent variable (tournament incentive) and other control variables. The sample distribution by industry is given in Appendix A. The study’s final sample is comprised of 6515 observations excluding firm-year observations for which the required information on explanatory variables were missing.

### 3.2. Measures

#### 3.2.1. Dependent Variable

Our study’s dependent variable is green innovation (GI), representing environmentally related patents. Consistent with previous studies on green innovation and the availability of data in the context of China, we selected a summary of green patents using several terminologies related to the environment, such as green, less carbon, environmental protection, clean, emission reduction, sustainable, recycling, economical, ecology, and environmental performance [112,113]. Eventually, we accumulated the patent number at the firm level and obtained each firms’ green innovation.

#### 3.2.2. Independent Variable

Our main independent variable is the tournament incentive (T_Incentives). Following previous studies [55,88,114,115], we measure CEO tournament incentives (T_Incentives) as the difference between CEO pay and the other executives of a firm. We applied the following distinctive formula to calculate the tournament incentive:

T_Incentives = Log (CEO Pay − Average Executives Pay)

In addition, we estimate the impact of CEO tournament incentives on green innovation in SOEs and non-SOEs. Hence, we measured SOE as a dummy variable that equals ‘1’ when the controlling shareholder of Chinese firms is the local or central government and ‘0’ otherwise.

#### 3.2.3. Control Variables

In line with previous studies on green innovation and CEO attributes [1,18,55,89,116,117], we control for a number of firm-level characteristics. CEO features include CEO age (CEO_Age), calculated in number of years. CEO duality is a dummy variable for which we assign one when a CEO also serves as a board chairman and zero otherwise. CEO ownership (CEO_Shares) is defined as the percentage of shares held by the CEO. This study also adds board ownership (Board_Shares), or the percentage of shares owned by a firm’s board members, and board size (Board_Size), measured as the number of directors on the board because board members also contribute to important strategic choices. A company’s financial attributes can also influence its societal engagements [50]. Thus, we include firm size (Size), measured as a log of total assets; firm size (Growth), calculated as the increase in a firm’s total assets; financial leverage (Leverage), which is the ratio of the total debt divided by the total assets; and firm performance (ROA), explains accounting-based performance measured as the net profit divided by the total assets. Finally, we include year and industry dummies in each regression analysis to capture the fixed effects of time and industry that may have an impact on the relationship between CEO tournament incentives and green innovation.

### 3.3. Statistical Model

To test the hypothesis of whether a firm’s tournament incentives inclined CEOs towards green innovation, we estimate Equation (1). To determine whether CEOs in state-owned enterprises are more motivated for green innovation as compared to non-state-owned enterprises, we apply Equation (1) for both the state and non-state firms’ samples. Consistent with previous studies [50,55,118], we apply an ordinary least squares (OLS) model as the baseline approach for estimating Equation (1). The model is as follows:

(1) $${\mathrm{GI}}_{\mathrm{it}}=\beta_{0}+{\;\beta }_{1}\;T\_{\mathrm{Incentives}}_{\mathrm{it}}+\overset{n}{\underset{i=1}{\sum }}\beta_{n\;}{\mathrm{Controls}}_{\mathrm{it}}+\varepsilon_{\mathrm{it}}$$

In the above model, GI indicates green innovation, representing the dependent variable. T_Incentives is our main independent variable, and controls refers to all control variables. Table 1 reports a detailed description of each variable.

### 3.4. Descriptive Statistics and Correlations

Table 2 illustrates the summary statistics for all variables used in the analysis. The results show that the mean GI score is 2.553, which indicates that, on average, Chinese firms’ secure three environment-related patents that reflects their green performance. A higher mean score of green innovation shows higher environmental performance. The mean of the tournament incentives (T_Incentives) is 402,665 Yuan (CNY), showing that CEOs receive an average 402,665 Yuan (CNY) annually beyond the pay that other executives receive. The mean value of SOEs is 0.467, which declares that 47 percent of the firms in the sample are state-owned enterprises where state owners are the major shareholders.

Table 3 presents the correlation for selected variables using a variance inflation factor (VIF) test and a Pearson’s correlation. The results show that the correlation between the green innovation and tournament incentive is positive and significant. We also find SOEs to be positively associated with green innovation; thus, the results provide preliminary support for our propositions. Additionally, the correlation between all independent variables is within the acceptable limits, which shows that there is no problem of multicollinearity.

## 4. Results

Table 4 displays the OLS regression results for the proposed hypotheses. Hypothesis 1 expects a significant and positive relation between CEO tournament incentives and green innovation. Model 1 of Table 4 lends support to the first hypothesis of this study by showing that the coefficient of CEOs tournament incentives (T_Incentives) is significantly positive (β = 0.165, *p* < 0.05). This result confirms that tournament incentives encourage CEOs to invest more in the firm’s green innovation to boost environmental performance. Moreover, the result supports tournament theory, which posits that the compensation gaps enhance competitiveness among CEOs and other top executives, thereby encouraging top executives to be greener by investing in environmentally related projects. This finding is consistent with latest study of Ali et al. [55], who documented a positive influence of CEO tournament incentives on CSR.

Table 4 also presents the findings for the sub-samples of state-owned and non-state-owned firms. Model 2 shows the finding of SOEs sub-sample, where the coefficient of the tournament incentive is 0.697, significant at 1%. The finding implies that top executives of state-owned firms receive tournament incentives so that they are more ingrained in environmental performance and earn legitimacy. The reason is that the CEOs of state-owned firms receive pressure from the government and public to meet political and social targets. The result also supports institutional theory [119], whichpostulates that organizations seeking legitimacy succumb to institutional pressure, thus influencing their behavior. Alternatively, Model 3 reports the coefficient of CEO incentives for non-SOEs which is 0.203, significant at 1%, indicating that CEO tournament incentives in non-state firms also have a positive impact on green innovation.

However, comparing the findings of state and non-state enterprises indicates that a state enterprise’s executive incentives have a greater and more favorable impact on green innovation than non-state firms, which acknowledges our second hypothesis. The coefficient value of Model 2 is significantly greater than that of Model 3. In order to compare the beta values for every model, we use an independent regression technique. Because of the significant influence of tournament incentives in state firms, the Chi^2^ value (9.601), which has a *p*-value = 0.001, shows that green innovation is more visible in state firms than in non-state firms.

Other robustness checks were also used to verify the authenticity of our conclusions. For the baseline regression findings, we attempted ordinary least squares (OLS) estimation, but the OLS criterion that observations should be independent could not be met in our analysis, as we use more than one observation in each of the selected companies, which could lead to biased estimates. To overcome this limitation, we used OLS regression with clustering of the standard error by firm (Cluster-OLS). Table 4 highlights the results for the cluster OLS estimations. These findings again confirm that tournament incentives positively affect corporate green innovation, and this relationship is stronger in state-owned firms.

Table 4 also highlights results for firm-level control variables. We find that except for CEO ownership, age, board ownership, and growth, all other variables have significant impacts on green practices. In particular, the coefficient of CEO duality indicates that it may generate a negative impact on environmental innovation. This is because a CEO may grow more powerful with dual job status, and, hence, may utilize corporate resources for self-interest rather than for societal interest. Conversely, Board_Size is positively associated with GI because a larger board contains the diversified knowledge and experience that often leads to firms going green. Similarly, firm size (Size) and profitability (ROA) have positive relationships with green innovation, as larger and more profitable firms receive more attention from stakeholders with regards to environmental concerns. In addition, such firms are considered resourceful to invest in green innovation. Finally, the coefficient of *leverage* suggests that with higher level of leverage, firms will be less likely to invest in green innovation.

## 5. Endogeneity

Our OLS regression results on the link between CEO tournament incentives and green innovation may be biased due to potential endogeneity issues. To solve this problem, our study uses three alternative statistical methodologies following previous research studies [55,113,120].

First, this study uses fixed-effects estimations to address the issue of unobserved factors (The Hausman test has been used to check the probability that a fixed-effects model or random effect is suitable to use. In doing so, the value of the Hausman test (247.66 at *p* < 0.01) confirms that the fixed-effects estimation is suitable over the random effect model.). For instance, one of the major issues in the estimation of panel datasets is the possibility of unobservable time-invariant characteristics. In such a case, a fixed-effects model has an advantage over other OLS estimations of explicit models’ characteristics, which are quite stable, thereby reducing cross-sectional fluctuations.

Table 5 presents the findings of the fixed-effect model and affirms that T_Incentives significantly and positively impacts a firm’s environmental performance. In addition, the findings of the sub-samples (SOEs and non-SOEs) suggest that tournament incentives in state firms increases the CEOs’ likelihood, more than in non-state firms, for investing in environmentally responsible projects. However, the regression models using fixed effects provide results that are consistent with the baseline analysis, confirming that our conclusion is unaffected by the omitted-variable bias.

Second, due to self-selection bias, our first OLS findings may be inaccurate, i.e., the features of a more environmentally responsible and a less environmentally responsible firm may differ, resulting in different outcomes for firms to be greener. In such a case, firms are considered greener because of firms-level characteristics, rather than due to increased CEO incentives. To address this issue, this study creates a dummy variable that equals one if a firm secures environmentally related patents more than the industry median, and zero otherwise, and compares the firms on the basis of the control variables by applying a propensity score matching (PSM) technique.

The findings of PSM models are highlighted in Table 6. However, consistent with our main evidence in Table 4, we find in all three models of Table 6, that T_Incentives are positively and significantly related to green innovation. In addition, we observe in Model 2 and Model 3 that the coefficient of T_Incentives remains greater for the SOEs’ sample, confirming that tournament incentives induce the SOEs’ CEOs more for investing in green innovation than the CEOs of NSOEs. Overall, these results suggest that our main findings are not driven by the firm-level characteristics.

Next, we apply an instrumental variable method known as Two-Stage least squares regression (2SLS) to address endogeneity issues. In this technique, the criteria that an “instrumental variable should be correlated with the independent variable but not with the dependent variable” must be fulfilled. Following this condition, our study uses the tournament incentives’ industry average as an instrumental variable because it is highly correlated with our independent variable (T_Incentives) but has no correlation with our dependent variable (GI). The results of the 2SLS method are reported in Table 7. Again, we find that these results are consistent with our initial findings, which makes our main evidence more robust.

## 6. Discussion and Conclusions

We consider the importance of a top executive’s role in environmental performance, as green practices cannot be initiated without top management support [20]. This research takes a holistic view to examine whether CEO tournament incentives result in improved environmental performance and fills the gap identified by [55]. Our expectations are founded on tournament theory, which states that promotions, awards, and incentives incentivize managers to accomplish company goals. These types of incentives create a competitive spirit among senior managers, who promote both financial and social performance within organizations [45].

When the executives choose to concentrate on consumers, workers, the environment, and other stakeholders, rather than shareholders, they have a great deal of flexibility [121]. It is established that compensation for non-financial performance is an approach by which a firm’s internal governance may affect its corporate environmental performance. Theoretically, a well-calculated prize range can stimulate prolific performance in a competition for incentives. It is argued that competing participants tend to perform better when the determination of compensation is carried out, keeping in mind the tournament viewpoint [44]. This indicates that if there is a pay disparity, a competition between the CEO and other managers will develop, resulting in better organizational effectiveness in terms of financial and non-financial performance, which greatly enhances the firm’s position in the marketplace. In line with this conventional notion, we argue that executives tournament incentives push them to spend more on environmentally friendly innovations, since such innovations may promote the reputation of an organization, stakeholders’ trust, brand image, and reduce tensions among the many stakeholders. Furthermore, as the CEOs positions rise, they are more inclined to focus on the company’s long-term success.

### 6.1. Theoretical Implications

This study used a sample of Chinese listed firms over the time period from 2010 to 2016 and confirms that CEOs are more inclined to invest in green innovation as a result of the tournament incentive. Our findings support tournament theory, suggesting that when compensation is determined on the basis of tournaments, the competing participants perform better. Compensation gaps encourage top decision makers to reach decisions with social, economic, and strategic consequences in mind. The pay disparity between the tournament winner and the runner-up acts as a strong motivator for senior managers to put greater efforts into winning the tournament. Organizations may gain from the aggregate enthusiasm of managers because pay incentives drive individual efforts to perform well [56,122]. Hence, our findings imply that firms’ green practices and their environmental legitimacy can be improved at the expense of incentivizing their executives. Top executives are considered powerful figures; thus the structuring of handsome compensation schemes for CEOs is advisable to stimulate a CEO’s likelihood to deploy efforts and resources towards green initiatives.

In addition, we find that when receiving tournament incentives in SOEs, executives are more engaged in green practices than in non-SOEs. These findings contribute to institutional theory, which advocates that, in a particular business environment, organizations need to imitate social means as the existence of the firm depends on external social approval [119]. Moreover, social and environmental behavior is contextualized by institutional pressures [123]. The SOEs face more stringent regulatory and institutional pressure (such as government and media) as compared to non-SOEs [50], which may induce SOEs further to adopt environmentally friendly policies. Neo-institutional theory also suggests that the institutional structure influences the decision making of a firm [124] and permits discovering and comparing executives’ non-financial motives (like green investment) in both formal and informal institutional contexts [125].

### 6.2. Limitations of the Study

Although, the study objectives are attained, certain limitations are essential to explain. These limitations should be considered for future potential research, as well as for the interpretation and generalizability of the results. First, our study sample is confined by the unique structure of the Chinese market, where most of the firms are controlled by the government. In this way, the generalization of the findings is constrained, as the Chinese market varies from those of advanced nations. Therefore, a future study could be carried out to verify the same arguments in developed countries. Second, patents are not the only way to safeguard green innovations; lead times, industrial capacity, and complicated standards can all have an impact [126,127]. Therefore, future research work can be conducted to capture the other dimensions of green innovations that will further determine the applicability of results. Finally, the study results assert that top executive tournament incentives push more state-owned firms towards environmental innovation as compared to non-government firms, but this relationship may be aroused through mediated factors, such as societal expectations or government pressure. The reason is that private firms take social objectives as a cost, not as holy obligations, while, the sense of responsibility to achieve societal targets and government supervision drives state organizations to pursue environmental stewardship. This concern must be addressed in future work through an exploration of the role of social- and state-level pressures on CEOs’ excellent performance in state-level organizations and how these influence their incentives and environmental innovation.

## Figures and Tables

**Table 1:** Variables definitions and details.

Variable	Details
GI	Green innovation is defined as the number of environment related patents (such as patents related to environmental protection, low carbon, eco-energy saving, emission reduction, sustainability, recycling, pollution, water resources, and biodiversity).
T_Incentives	Log of the CEO compensation gap and the average pay of other executives.
SOE	Defined as the dummy variable which equals one if the local or central government is the ultimate owner and zero otherwise.
CEO_Shares	Indicates the percentage of shares owned by a CEO.
CEO_Age	Equals age of the CEO in years.
CEO_Duality	A dummy variable equals one if the CEO is also the chairperson of the board and zero otherwise.
Board_Shares	The percentage of shares held by a firm’s board of directors.
Board_Size	Defined as number of directors on the board.
Size	Indicates the natural log of total assets.
Growth	Calculated as change in total assets.
Leverage	Indicates ratio of total debt to total assets.
ROA	Shows return on assets, which is measured by dividing the net profit by total assets.

**Table 2:** Descriptive statistics.

Variable	Mean	Std. Dev.	Min	Max
GI	2.553	5.23	0	39
T_Incentives	402,665	545,034	−915,000	1,500,000
SOE	0.467	0.498	0	1
CEO_Shares	0.041	0.107	0	0.805
CEO_Age	48.588	6.229	25	77
CEO_Duality	0.246	0.431	0	1
Board_Shares	0.103	0.186	0	0.892
Board_Size	10.211	2.557	5	26
Size	3.090	0.058	2.704	3.350
Growth	7.665	1.336	1.609	13.223
Leverage	0.456	0.562	0.007	46.159
ROA	0.039	0.545	−48.316	22.005

**Table 3:** VIF test and Pearson’s correlation.

Variables	VIF	(1)	(2)	(3)	(4)	(5)	(6)	(7)	(8)	(9)	(10)	(11)	(12)
(1) GI	--	1.000											
(2) T_Incentives	1.13	0.064 *	1.000										
		(0.000)											
(3) CEO_Shares	2.47	−0.040 *	−0.081 *	1.000									
		(0.001)	(0.000)										
(4) CEO_Age	1.13	0.061 *	0.140 *	−0.004	1.000								
		(0.000)	(0.000)	(0.711)									
(5) CEO_Duality	1.51	−0.029 *	0.021 *	0.475 *	0.129 *	1.000							
		(0.013)	(0.039)	(0.000)	(0.000)								
(6) Board_Share	2.56	−0.055 *	−0.132 *	0.698 *	−0.118 *	0.257 *	1.000						
		(0.000)	(0.000)	(0.000)	(0.000)	(0.000)							
(7) Board_Size	1.15	0.082 *	0.117 *	−0.133 *	0.043 *	−0.104 *	−0.168 *	1.000					
		(0.000)	(0.000)	(0.000)	(0.000)	(0.000)	(0.000)						
(8) Growth	1.04	−0.017	0.012	0.050 *	−0.028 *	0.048 *	0.064 *	0.049 *	1.000				
		(0.152)	(0.228)	(0.000)	(0.004)	(0.000)	(0.000)	(0.000)					
(9) Size	1.35	0.002	−0.008	−0.002	−0.009	−0.004	−0.005	0.019 *	0.179 *	1.000			
		(0.893)	(0.452)	(0.810)	(0.344)	(0.715)	(0.621)	(0.049)	(0.000)				
(10) Leverage	1.22	0.065 *	0.012	−0.102 *	−0.012	−0.042 *	−0.150 *	0.066 *	−0.031 *	0.004	1.000		
		(0.000)	(0.243)	(0.000)	(0.238)	(0.000)	(0.000)	(0.000)	(0.001)	(0.713)			
(11) ROA	1.01	−0.002	0.029 *	0.013	0.017	−0.004	0.018	−0.020 *	0.063 *	0.000	−0.738 *	1.000	
		(0.834)	(0.004)	(0.210)	(0.076)	(0.678)	(0.056)	(0.040)	(0.000)	(0.996)	(0.000)		
(12) SOE	1.60	0.078 *	0.068 *	−0.339 *	0.144 *	−0.296 *	−0.493 *	0.217 *	−0.079 *	0.009	0.113 *	−0.011	1.000
		(0.000)	(0.000)	(0.000)	(0.000)	(0.000)	(0.000)	(0.000)	(0.000)	(0.345)	(0.000)	(0.270)	

* shows significance level at 5%.

**Table 4:** Main evidence of CEOs tournament incentives and green innovation.

	OLS Regressions			Cluster-OLS Regressions		
Variables	Full Sample	SOEs	N-SOEs	Full Sample	SOEs	N-SOEs
T_Incentives	0.165 **	0.697 ***	0.203 ***	0.613 ***	0.711 ***	0.681 ***
	(2.465)	(5.128)	(2.666)	(4.798)	(3.362)	(3.087)
CEO_Shares	0.145	−15.515	0.330	1.183	1.095	−15.582
	(0.185)	(−1.181)	(0.458)	(0.972)	(0.931)	(−1.163)
CEO_Age	0.001	0.052	0.007	0.019	−0.016	0.053 *
	(0.136)	(0.466)	(0.684)	(1.328)	(−1.245)	(1.732)
CEO_Duality	−0.100 **	−0.365 *	−0.073 *	−0.574 **	−0.352	−0.376
	(−2.148)	(−1.741)	(−1.813)	(−2.267)	(−1.402)	(−0.619)
Board_Shares	0.609	−6.760	0.286	−1.747 ***	−0.995	−6.893
	(1.394)	(−0.955)	(0.686)	(−2.599)	(−1.573)	(−0.980)
Board_Size	0.011 *	0.059 **	0.058 *	0.076 *	0.099 **	0.061
	(1.765)	(2.378)	(1.939)	(1.839)	(2.203)	(0.867)
Growth	−0.000	−0.151	−0.009	0.011	0.033	−0.050
	(−0.009)	(−1.486)	(−0.214)	(0.260)	(0.950)	(−0.562)
Size	1.691 ***	0.023 **	1.193 ***	2.121 **	1.473	3.473 **
	(31.997)	(2.335)	(17.381)	(2.236)	(1.491)	(2.278)
Leverage	−0.699 ***	−3.480 ***	−0.743 ***	0.011	−0.017	−6.774 **
	(−3.177)	(−6.586)	(−3.157)	(0.080)	(−0.217)	(−2.206)
ROA	0.348 *	7.119 ***	0.231 *	1.044 ***	5.500 **	3.931 ***
	(1.687)	(2.910)	(1.784)	(2.776)	(2.718)	(3.105)
Constant	−14.609 ***	−13.616 ***	−10.849 ***	−9.094 ***	−5.516 **	−13.571 ***
	(−13.463)	(−6.270)	(−8.743)	(−4.606)	(−2.368)	(−4.185)
Observations	6515	2740	3775	6515	2740	3775
R-squared	0.223	0.142	0.149	0.100	0.081	0.140
Industry & Year	Yes	Yes	Yes	Yes	Yes	Yes
Chi^2^ value = 9.600 *p*-value = 0.001						

Note: Table 4 reports result for the proposed hypotheses. T_Incentives indicate tournament incentives of CEOs. T-statistics are documented in parentheses. ***, **, and * denote significance level at 1, 5, and 10%, respectively. This table also reports the value of Chi^2^ for the sake of sub-sample comparison.

**Table 5:** Endogeneity test: fixed-effect model.

Variables	Full Sample	SOEs	N-SOEs
T_Incentives	0.264 ***	0.292 ***	0.236 *
	(3.079)	(2.699)	(1.657)
CEO_Shares	−0.582	1.623	−0.771
	(−0.555)	(0.060)	(−0.741)
CEO_Age	0.030 **	0.053 **	0.010
	(2.298)	(2.454)	(0.605)
CEO_Duality	−0.206	−0.474	−0.006
	(−1.036)	(−1.358)	(−0.024)
Board_Shares	0.522	20.592 *	0.060
	(0.506)	(1.758)	(0.059)
Board_Size	0.011	0.015	−0.006
	(0.454)	(0.393)	(−0.178)
Growth	−0.092 ***	−0.052	−0.121 ***
	(−3.001)	(−1.065)	(−3.182)
Size	0.510 ***	0.334 ***	0.798 ***
	(4.252)	(2.815)	(4.663)
Leverage	0.020	−2.086 *	−0.775
	(0.059)	(−1.902)	(−1.157)
ROA	0.116	−2.168	0.040
	(0.767)	(−1.024)	(0.227)
Constant	−1.128 ***	−6.453 ***	−1.494 ***
	(−2.770)	(−2.711)	(−2.819)
Observations	6515	2740	3775
R-squared	0.089	0.110	0.081
Year	Yes	Yes	Yes

Note: Table 5 presents findings of fixed-effect model, which addresses issues of omitted factors. T-statistics are documented in parentheses. ***, **, and * denote significance level at 1, 5, and 10%, respectively.

**Table 6:** Propensity score matching (PSM) results.

Variables	Full Sample	SOEs	N-SOEs
T_Incentives	0.1444 ***	0.1548 ***	0.0830 ***
	(7.75)	(5.15)	(3.26)
CEO_Shares	0.4137 *	−1.4760	0.1738
	(1.80)	(−0.49)	(0.71)
CEO_Age	0.0059 **	0.0123 ***	0.0016
	(2.22)	(2.71)	(0.48)
CEO_Duality	−2334 ***	−2532 ***	−1213 **
	(−5.35)	(−3.25)	(−2.18)
Board_Shares	−5153 ***	0.1516	−1162
	(−4.19)	(.10)	(−0.84)
Board_Size	0.0258 ***	0.0247 ***	0.1222
	(3.96)	(2.76)	(1.22)
Size	0.0056	−0271	0.02551 **
	(0.47)	(−1.22)	(1.97)
Growth	0.0023	0.0035	0.2197 ***
	(1.12)	(1.12)	(9.63)
Leverage	−3348 ***	−4381 ***	−1596 **
	(−6.33)	(−4.26)	(−2.28)
ROA	−0939	−0375	−2039
	(−0.52)	(−0.07)	(−0.51)
Constant	−2.7491 ***	−3.159 ***	−3.3937 ***
	(−10.59)	(−7.57)	(−9.44)
Observations	6515	2740	3757
R-squared	0.0315	0.0214	0.0412
Industry & Year	Yes	yes	Yes

Note: Table 6 highlight results to validate our main hypotheses using PSM approach. T-statistics are documented in parentheses. ***, **, and * denote significance level at 1, 5, and 10%, respectively.

**Table 7:** Two Stage least squares (2SLS) results.

Variables	Full Sample	SOEs	N-SOEs
T_Incentives	1.002 ***	1.285 ***	0.368 ***
	(7.712)	(5.384)	(2.618)
CEO_Shares	1.692	−9.922	0.436
	(1.472)	(−0.544)	(0.445)
CEO_Age	0.011	0.039	−0.016
	(0.763)	(1.436)	(−1.124)
CEO_Duality	−0.830 ***	−0.761 *	−0.198
	(−3.806)	(−1.767)	(−0.880)
Board_Shares	−1.733 ***	−14.071	0.538
	(−2.713)	(−1.443)	(0.942)
Board_Size	0.074 **	0.031	0.077 *
	(2.210)	(0.601)	(1.885)
Growth	−0.101	−0.225	−0.068
	(−1.321)	(−1.636)	(−0.867)
Size	0.022 **	0.028 **	1.361 ***
	(2.488)	(2.449)	(4.505)
Leverage	−2.633 ***	−7.014 ***	−0.847 ***
	(−8.400)	(−8.112)	(−3.021)
ROA	2.912 *	8.587 ***	1.370
	(1.781)	(2.715)	(0.807)
Constant	−11.560 ***	−18.606 ***	−12.490 ***
	(−6.512)	(−5.784)	(−6.681)
Observations	4596	2044	2547
R-squared	0.105	0.155	0.157
Industry & Year	Yes	Yes	Yes

Note: Table 7 presents results of instrumental variable approach (2SLS). T-statistics are documented in parentheses. ***, **, and * denote significance level at 1, 5, and 10%, respectively.

## Data Availability

The data that support the findings of this study are available on reasonable request from the corresponding author.

## Appendix A

**Table A1:** Sample Distribution by Industry (*N* = 6515).

Industry Name	Full Sample	SOE Obs	N-SOE Obs
Agriculture, forestry, animal husbandry, and fishery	75	29	46
Mining industry	279	97	182
Manufacturing industry	4181	1593	2588
Electricity, thermal, gas and water production, and supply industry	218	163	55
Construction business	225	102	123
Wholesale and retail business	295	178	117
Transportation, warehousing, and postal service	206	173	33
Accommodation and catering	72	28	44
Information transmission, software and information technology services	428	132	296
Real estate	172	96	76
Culture, sports and entertainment	33	10	23
Leasing and business services	41	13	28
Scientific research and technology services	46	18	28
Water conservancy, environment and public facilities management	34	11	21
Education	58	24	34
Health and social work	115	46	69
Comprehensive	37	25	12
